# In This Issue: Volume 117, Issue 10, October 2026

**DOI:** 10.1111/cas.70526

**Published:** 2026-10-02

## Genetic Evolution in *BRCA1* ‐Associated Breast Cancer Reveals Early Driver Mutations Shaping Tumor Features and Prognosis

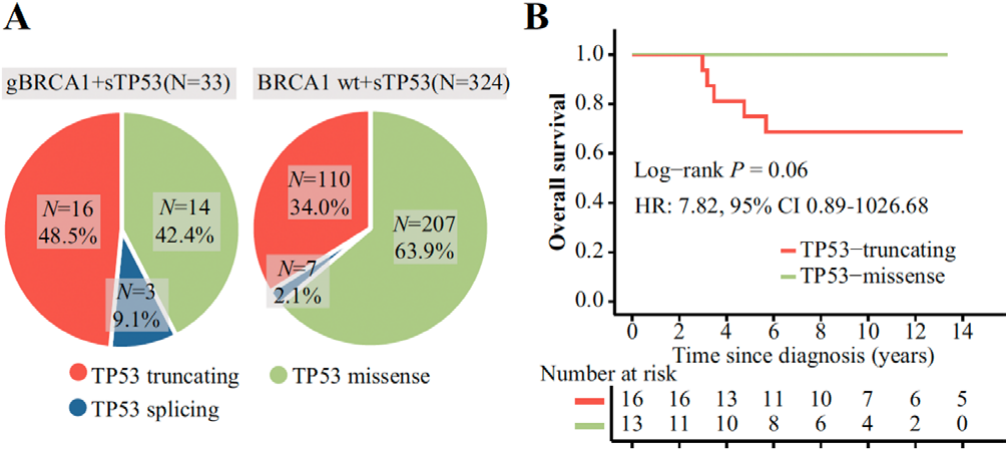

Breast cancer develops when cells acquire genetic changes that allow them to grow and survive abnormally. People who inherit harmful changes in the BRCA1 gene have a higher risk of breast cancer, often at a younger age. Their tumors are also more likely to be triple‐negative breast cancers (TNBCs), meaning they lack three common markers used to guide treatment. However, it has been unclear which genetic changes occur early in *BRCA1*‐related breast cancer and how these early changes influence tumor characteristics and outcomes.

The researchers studied 57 primary breast tumors from 57 people with harmful inherited *BRCA1* variants. They examined tumor samples from different stages, including carcinoma in situ, an early form of cancer that has not yet invaded surrounding tissue, and invasive cancer. Genetic testing was used to identify cancer‐driving changes and estimate when they appeared during tumor development. *TP53*, a gene that normally helps prevent damaged cells from growing, was the most common early driver, initiating about 58% of the tumors. Mutations in this gene overall were found in about 70% of the tumors. When *TP53* was the first major genetic change, tumors were usually triple‐negative (about 88%) and were more likely to have loss of the remaining normal *BRCA1* copy and higher levels of genomic instability.

Together, these findings show that the genetic changes that occur early in *BRCA1*‐related breast cancer can help shape the type and behavior of the resulting tumor. The researchers also found that *TP53* truncating mutations (nonsense or frameshift), which prevent the gene from producing a complete protein, were significantly more common in *BRCA1*‐related tumors than in other breast cancers. These mutations were linked to lower homologous recombination deficiency (HRD) scores, activation of epithelial‐mesenchymal transition (EMT) pathways, and a trend toward poorer survival outcomes. Because the study included a relatively small number of patients, these findings need to be confirmed in larger groups. Still, understanding when and how these genetic changes occur could help researchers better understand aggressive BRCA1‐related tumors and may support future approaches to risk assessment and treatment.

https://onlinelibrary.wiley.com/doi/full/10.1111/cas.70460

## FDX1 Depletion Activates CD8 ^+^ T Cell Antitumor Immunity by Promoting DMBT1 Secretion in Cuproptosis of Colorectal Cancer

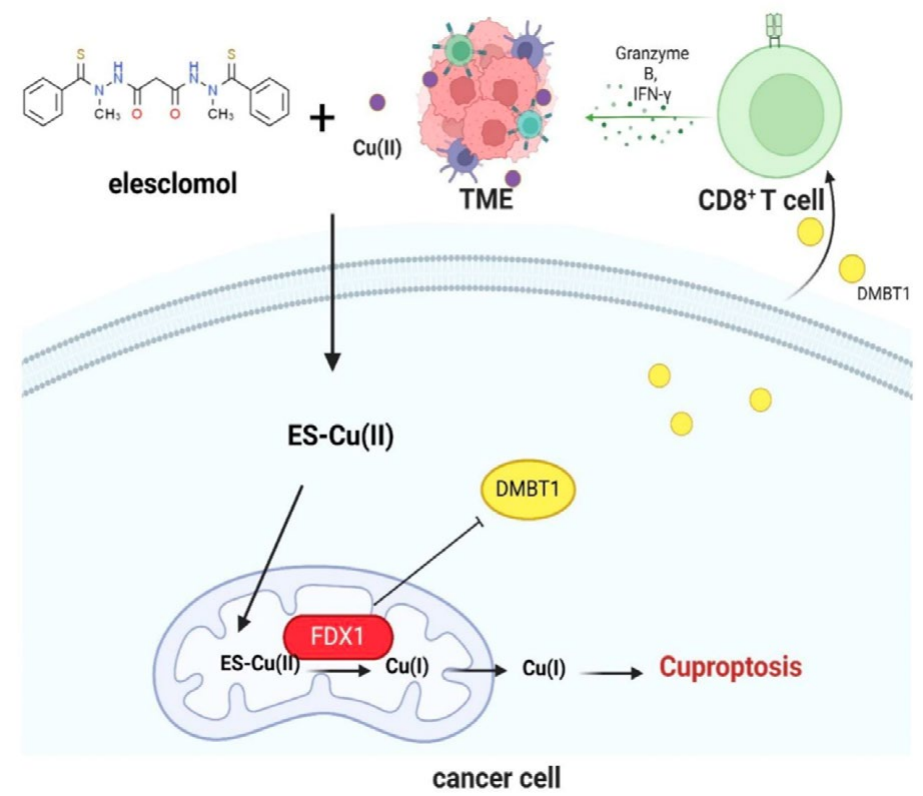

Colorectal cancer (CRC) is one of the most common cancers in the world and significantly contributes to cancer‐related death. Although the immune system can recognize and attack cancer cells, tumors may develop ways to weaken this response. In particular, CD8+ T cells, which can directly kill cancer cells, may become less effective or “exhausted” within the tumor environment. Identifying ways to strengthen these immune cells could improve treatment outcomes for patients with colorectal cancer.

In this study, Wu et al. explored a process known as cuproptosis, a copper‐dependent form of regulated cell death. They focused on FDX1, a molecule that plays an important role in cuproptosis. The researchers found that inhibiting FDX1 in CRC cells not only slowed cancer cell growth but also improved the ability of CD8+ T‐cells to attack the tumor.

The researchers also found that FDX1 controls the production and release of a molecule known as DMBT1. When FDX1 was inhibited, cancer cells produced and secreted higher amounts of DMBT1. This, in turn, helped CD8+ T‐cells remain active and better equipped to fight against cancer cells, with increased production of cancer‐destroying molecules such as granzyme B and perforin and reduced signs of T‐cell exhaustion.

It was also observed that another molecule, DICER1, which controls the stability of genetic messages inside cells, plays an important role in how FDX1 influences DMBT1. This finding helped uncover a connected FDX1‐DICER1‐DMBT1 pathway linking internal changes in cancer cells to immune cell activity.

The researchers validated their findings in cancer cells, mouse models, and human CRC samples. Higher levels of FDX1 were associated with poorer outcomes and reduced CD8+ T‐cell activity, while increased DMBT1 was associated with a stronger antitumor response.

These findings suggest that FDX1 can influence both CRC progression and the body's immune response against the tumor. As a result, targeting the FDX1‐DICER1‐DMBT1 pathway may provide a potential approach to enhance CD8+ T‐cell‐based antitumor immunity. However, further research is required to confirm whether this strategy can be safely and effectively developed into a treatment for patients.

https://onlinelibrary.wiley.com/doi/full/10.1111/cas.70457

## Selective Depletion of Tumor‐Infiltrating CD4 ^+^ CCR8 ^+^ Tregs by AZ084, a Novel CCR8 Antagonist

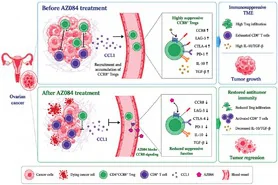

Ovarian cancer can be difficult to treat, particularly when diagnosed at an advanced stage. One reason is the tumor's ability to weaken the immune response. Regulatory T cells (Tregs), which normally help prevent excessive immune reactions, can accumulate inside tumors and suppress immune cells that could otherwise attack cancer. Finding ways to target these Tregs without disrupting immune function elsewhere could therefore improve cancer treatment.

In this study, Mao et al. focused on CCR8, a protein found at high levels on a group of Tregs within tumors. In mouse models of ovarian cancer and melanoma, CCR8‐positive Tregs showed stronger immune‐suppressing activity than other Treg populations. The researchers then tested AZ084, a drug that blocks CCR8. In laboratory experiments, AZ084 reduced Treg differentiation, weakened the ability of CCR8‐positive Tregs to suppress immune cells, and reduced their migration toward tumor‐associated signals.

In tumor‐bearing mice, AZ084 treatment reduced tumor growth and decreased the number of CCR8‐positive Tregs within tumors. At the same time, more CD8‐positive T cells entered the tumors and showed increased production of interferon‐gamma, a molecule involved in the immune response against abnormal cells. Importantly, AZ084 caused minimal changes in major immune cell populations in the spleen and did not produce clear signs of organ damage under the conditions tested.

These findings suggest that blocking CCR8 may help reduce immune suppression within tumors while preserving immune balance elsewhere. However, the study was conducted mainly in mouse models, and further research is needed to understand the mechanism, evaluate safety, and determine whether AZ084 could be effective in people.

https://onlinelibrary.wiley.com/doi/full/10.1111/cas.70465

